# Filiform Polyposis in Ulcerative Colitis: A Rare Pediatric Case

**DOI:** 10.4274/balkanmedj.2018.0136

**Published:** 2018-09-21

**Authors:** Nafiye Urgancı, Derya Kalyoncu, Canan Tanık

**Affiliations:** 1 Clinic of Pediatric Gastroenterology, İstanbul Şişli Hamidiye Etfal Training and Research Hospital, İstanbul, Turkey; 2 Clinic of Pediatrics, İstanbul Şişli Hamidiye Etfal Training and Research Hospital, İstanbul, Turkey; 3 Clinic of Pediatrics, İstanbul İstinye State Hospital, İstanbul, Turkey; 4 Department of Patology, University of Health Sciences, Şişli Etfal Training and Research Hospital, İstanbul, Turkey

**Keywords:** Children, filiform, polyposis, ulcerative colitis

## Abstract

**Background:**

Filiform polyposis is a rare benign condition referred to as inflammatory polyposis, or pseudopolyposis that is usually found in association with Crohn’s disease, ulcerative colitis or granulomatous colitis which is formed by non-specific mucosal and submucosal reactions to previous severe inﬂammation. It is characterized by multiple finger-like projections most commonly in the transverse and descending colon.

**Case Report:**

A 15-year-old girl with a history of ulcerative colitis was admitted to the pediatric emergency department with abdominal pain attacks for the past 2 weeks. Abdominal ultrasound and magnetic resonance enterography revealed mucosal thickening in the transverse and descending colon. Colonoscopy revealed small filiform polyps throughout the colon. Histopathological examination revealed inflammatory polyps associated with ulcerative colitis.

**Conclusion:**

Non-neoplastic filiform polyps can be detected even in children with ulcerative colitis with long-term remissions.

Filiform polyposis is a rare form of pseudopolyposis associated with ulcerative colitis, Crohn’s disease, or granulomatous colitis (1,2). It has been reported that filiform polyps develop due to prolonged severe mucosal and submucosal inflammation in the colon of patients with inflammatory bowel disease (1,3). Finger-like polyps are characterized by the endoscopic appearance of multiple stalactites resembling villous adenomas in the colon (1). The polyps can be observed in all parts of the colon but commonly in the transverse and descending colon (2).

Although adult cases of filiform polyposis have been reported in the literature (1,3,4,5), there are only a few pediatric cases of inflammatory bowel disease with filiform polyposis (6,7). We report a case of a 15-year-old girl with ulcerative colitis who had filiform polyposis.

## CASE PRESENTATION

A 15-year-old girl with ulcerative colitis who was in remission and administered 5-aminosalicylic acidand intermittent prednisolone treatment for the past 6 years was admitted to the pediatric emergency department with abdominal pain attacks for the past 2 weeks. Her parents were first-degree cousins. She had a healthy sibling. She was born via spontaneous vaginal delivery with a birth weight of 3.200 g. She had no family history of inflammatory bowel disease and chronic or autoimmune disease.

Her physical examination revealed a body weight of 49 kg (10-25^th ^centile) and a height of 160 cm (25-50^th^ centile). She had fever of 36 °C. She was pale and had mild abdominal tenderness. The cardiovascular and respiratory system examinations were unremarkable.

The laboratory examinations revealed the following results: hemoglobin 10.5 g/L, white blood cell count 8000/mm^3^, platelet count 216.000/mm^3^, total protein 5 g/L, albumin 2.7 g/L, iron 24 ng/L, iron-binding capacity 380 ng/dL, ferritin 13 ng/mL, C-reactive protein 0.6 mg/dL, and erythrocyte sedimentation rate 11 mL/h. Urinary tests and stool analysis were normal.

**Figure 1:**
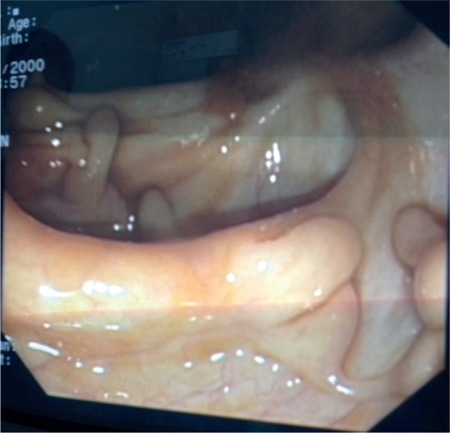
Endoscopic appearance of filiform polyps in the colon.

**Figure 2:**
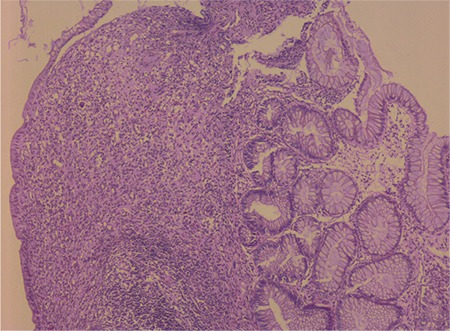
Ulcerated active granulation tissue, hyperplastic changes in crypts, crypt distortion, polypoid lesions including polymorphonuclear leukocytes, lymphocytes and plasma cells in the lamina propria (H&E x100).

Abdominal ultrasound and magnetic resonance enterography revealed mucosal thickening in the transverse and descending colon. Colonoscopy was repeated, which revealed small filiform polyps with 0.5×1.5×3 cm dimensions throughout the colon but most common in the rectosigmoid, descending, and transverse colon (Figure 1). Histopathological examination revealed inflammatory polyps associated with ulcerative colitis (Figure 2). Informed consent was obtained from the patient’s parents.

## DISCUSSION

Inflammatory filiform polyps in the colon of patients with ulcerative colitis  have been first reported by Appelman et al. (8) in 1974. The incidence of inflammatory polyps has been reported to be 10%-20% in both ulcerative colitis  and Crohn’s disease (5). It has been postulated that these polyps develop in patients with ulcerative colitis  and Crohn’s disease due to regeneration of mucosa during remission after recurrent acute inflammation (5). Moreover, these polyps have been determined as the asymptomatic sequela of ulcerative colitis  in clinicopathologic and immunophenotypic examinations (1). Rarely, these filiform polyps may be relatively large in size and are known as giant filiform polyps (3).

The cases reported in the literature were primarily young adults and elders (aged >38 years) (1,3,4,5). A limited number of pediatric cases have also been reported (6,7).

Typically, patients are admitted with anemia, weight loss, abdominal pain, diarrhea, and bleeding (1,3). Our patient was admitted with abdominal pain when she was in remission. She underwent colonoscopy for the suspicion of disease activation, which revealed filiform polyps throughout the colon. Histopathological examination of biopsy specimens revealed inflammatory polyps, without mucosal dysplasia.

Filiform polyps can bridge and fill the colon lumen. Widespread, long, and large-sized polyps can cause complications such as obstruction and bleeding (5). These polyps are non-neoplastic and are not an indication for colectomy. Since the patient’s disease was inactive, treatment continued. The patient and the parents were informed about the complications of filiform polyps and the patient was followed up closely.

In conclusion, non-neoplastic filiform polyps can be detected even in children with ulcerative colitis with long-term remissions.
